# Surgical Management of Giant Prolactinomas: A Descriptive Study

**DOI:** 10.1155/2023/1990259

**Published:** 2023-04-25

**Authors:** Michelle D. Lundholm, Divya Yogi-Morren, Kevin M. Pantalone, Pablo F. Recinos, Varun R. Kshettry, Pratibha P. R. Rao

**Affiliations:** ^1^Department of Endocrinology, Diabetes and Metabolism, Cleveland Clinic, Cleveland, OH, USA; ^2^Department of Neurological Surgery and Rose Ella Burkhardt Brain Tumor and Neuro-Oncology Center, Cleveland Clinic, Cleveland, OH, USA

## Abstract

**Introduction:**

Giant prolactinoma (GP) is a rare pituitary lactotropic cell tumor larger than 4 cm in its widest dimension, and is less likely than a smaller prolactinoma to achieve prolactin normalization on dopamine agonist (DA) monotherapy. There is a paucity of data on the circumstances and outcomes of second-line management of GP with surgery. Herein, our institution's experience with the surgical management of GPs is described.

**Methods:**

A single-center retrospective analysis was conducted of patients who underwent surgery for giant prolactinoma from 2003 to 2018. A chart review was conducted for demographic data, clinical features, laboratory and radiographic findings, operative and pathology reports, perioperative management, and clinical outcomes in follow-up. Descriptive statistics were used.

**Results:**

Of 79 prolactinoma cases, 8 patients had GP with a median age of 38 years (range 20–53), 75% (6/8) were male, with a median largest tumor dimension of 6 cm (range 4.6–7.7), and a median prolactin level of 2,500 *μ*g/L (range 100–>13,000). Six patients had transsphenoidal surgery for dopamine agonist (DA) resistance or intolerance. Two patients had a craniotomy for a missed diagnosis; one was due to the hook effect. No tumor resections were complete by either surgical approach; all had persistent hyperprolactinemia requiring postoperative DA therapy, and two patients had an additional craniotomy procedure for further tumor debulking. There was no recovery of pituitary axes and postoperative deficits were common. Remission as defined by prolactin normalization occurred in 63% (5/8) at a median time of 36 months (range 14–63 months) on DA therapy after surgery with a follow-up of 3–13 years.

**Conclusions:**

GPs infrequently require surgical resection, which is generally incomplete and requires adjuvant therapy. Given the rarity of surgery for GPs, multi-institutional or registry studies would yield clearer guidance on optimal management.

## 1. Introduction

Giant prolactinomas (GPs) are rare lactotropic tumors greater than 4 cm associated with markedly elevated serum prolactin (PRL) levels, classically in excess of 1,000 *μ*g/L. While generally benign, GPs tend to be invasive with suprasellar extension and compression of the optic chiasm. They can also distort the hypothalamus, compress the ventricular system, extend into the sphenoid, ethmoid, or cavernous sinuses, or erode into the skull base. Given the size of these tumors, GPs are more likely to cause mass effects including headaches, vision loss, or other neurologic changes. With radiographic and clinical features that are atypical of a pituitary tumor, the diagnosis may be more challenging to recognize and GPs may be mistaken for other invasive brain tumors such as gliomas, meningiomas, chordomas, or metastatic carcinomas.

Unlike other brain tumors, first-line treatment for prolactinomas is medical therapy with dopamine agonists (DAs). The goals of treatment include PRL normalization and tumor size reduction with relief of the mass effect. Initial tumor size is a main factor in predicting DA treatment success: PRL normalization is achieved in 90% of patients with prolactinoma on DAs alone but decreases to 70–80% for macroprolactinomas and 60–68% for GPs [1–3]. Cases which are refractory to DAs may try second-line therapies which include neurosurgery, radiation, or other medications such as somatostatin analogues or the alkylating cytotoxic agent temozolomide.

Surgical intervention is part of the management of approximately 13-14% of GPs [3]. Besides DA resistance, other indications for surgical resection of prolactinoma include DA intolerance, cerebrospinal fluid (CSF) leak, acute onset of severe visual or neurological symptoms, or pituitary tumor apoplexy. In several of these acute surgical indications, the primary goal is tumor decompression or leak repair. Surgery may additionally be considered for predominantly cystic tumors since they are less likely to shrink from medical therapy alone [4, 5], for women of child-bearing age to avoid tumor expansion and limit DA use in pregnancy, or by patient preference to avoid costs or risks of long-term DA use [6–8]. The decision to pursue an elective surgery must consider the risks of surgical complications such as diabetes insipidus, hypopituitarism, visual or mental deterioration, vascular arterial injury, cranial neuropathy, CSF leak, or meningitis. Complications occur in <5% of all prolactinoma operations, but risks increase with resection of larger and more invasive tumors [9–11].

Surgical cure rates (defined as long-term prolactin normalization off DA therapy) are relatively high for microprolactinomas (75–90%) and macroprolactinomas (45–70%) [8, 12–14]. However, postsurgical remission rates are lower in cases with markedly elevated preoperative prolactin levels, larger tumor size, extrasellar extension, or cavernous sinus invasion [15–20]. As all these features are common in GP tumors, it is unsurprising that limited observational data describe a 0% chance of complete resection or cure after a single operation for GP [6, 21–25]. Eventual prolactin normalization on DA postoperatively was achieved in 43–57% of reported cases in follow-up [23, 26]. The role of surgical management as part of a multimodal approach to the treatment of GP remains an area of investigation.

In this study, we describe our institution's understanding and experience with surgical intervention for GP. Our goal is to examine the circumstances which led to surgery and the clinical outcomes to better inform our future care of patients with this rare and challenging diagnosis.

## 2. Methods

A retrospective analysis was conducted on consecutive patients who underwent surgery for pathology-proven functional prolactinomas at the Cleveland Clinic between 2003 and 2018. Patients with GP were identified by a tumor size >4 cm on preoperative imaging. From the total of 1,080 patients in our operative series, there were 79 patients with prolactinoma, of which 8 patients (10%) had GP and were included in the present study. The chart review was performed for demographic data, clinical features, laboratory and radiographic findings, operative and pathology reports, perioperative management, and clinical outcomes in follow-up.

An ICD search of prolactinoma in our electronic medical record identified 1,487 prolactinoma cases from 2003 to 2018. Given that 2-3% of all prolactinoma are GP, it is estimated that there were approximately 22–37 cases of GP treated nonsurgically (30–45 total) at our center in the same time frame.

From May of 2014 to May of 2018, prolactin was measuring using a Roche Diagnostics Cobas assay, which does not have a hook effect up to a level of 12,690 *μ*g/L. Prior to May of 2014; our institution used the Siemens ADVIA Centaur prolactin test with an undetermined threshold for hook effect.

We defined DA resistance as persistent hyperprolactinemia (>15.2 *μ*g/L) or failure to reduce tumor size by ≥50% on a minimum of cabergoline 2.0–3.0 mg/week or bromocriptine 15 mg/day, or as tolerated, for at least 3 months of therapy [27, 28]. Patients were additionally considered resistant to DA if there was evidence of marked tumor growth or worsening of visual symptoms despite any duration of DA therapy. Biochemical remission was defined as PRL normalization (≤15.2 *μ*g/L) and a lack of hyperprolactinemia symptoms, with or without ongoing treatment.

Categorical variables are summarized using counts and percentages, and continuous variables are presented with a median and range. This study was approved by our Institutional Review Board.

## 3. Results

Eight patients with surgically-treated giant prolactinoma were identified; they were predominantly male (6/8, 75%) with median age of 38 years (range 20–53 years) at diagnosis. A summary of baseline characteristics is presented in Table 1. All patients presented with mass effects: 75% with headache, 86% with vision loss, 50% with ophthalmoplegia, and 50% with neurocognitive defect or behavioral change (including abulia, disinhibition, mood disturbance, emotional lability, and confusion). The median largest dimension of the tumor prior to any treatment was 6.0 cm (range 4.6–7.7 cm). The median tumor volume by the geometric formula was 31.0 cm^3^ (range 22.8–109.3 cm^3^). All giant tumors had optic chiasm compression and cavernous sinus invasion, and 2/8 (25%) had a cystic component (case numbers 1 and 3). Of the 7 patients who had preoperative testing, the median PRL level was 2,498 *μ*g/L (range 100–>13,000 *μ*g/L) prior to DA therapy. There was evidence of central hypogonadism in 6/6 patients (100%), and central hypothyroidism and adrenal insufficiency in 2/6 (25%).

Preoperative and operative treatment courses are summarized in Table 2. First-line DA therapy was attempted in 6 patients with GP on a median dose of cabergoline 2.5 mg/week (range 1.0 mg to 3.5 mg) for a median of 6 months (range 0.5–36 months). PRL levels decreased in all but one patient, (range 93% decrease to 19% increase), but none normalized. The largest dimension of the tumor decreased by a median of 2% (range 35% decrease to 30% increase), and tumor volume decreased by median of 11% (range 49% decrease to 44% increase). Of those patients who were medically treated preoperatively, the indication for surgery was based on DA resistance in 5/6 cases and intolerance (e.g. CSF leak and psychiatric symptoms) in 4/6 cases. There was additionally worsening vision loss in 5/6 and tumor apoplexy in 1/6. These patients had transsphenoidal resection with endoscopy or microscopy.

Two patients did not receive preoperative dopamine agonist therapy because prolactinoma was not diagnosed until the postoperative period. In one case, the diagnosis was missed due to the hook effect. These patients had craniotomies rather than transsphenoidal resection. By either surgical approach, no tumor resections were complete. All tumors were benign with diffuse prolactin staining. Two tumors co-stained, one with ACTH, and the other with GH and TSH, but neither corresponded to biochemical excess.

The postoperative treatment course for GP patients is presented in Table 3. All patients with GP had persistence of hyperprolactinemia at 1 week and 3 months postoperatively requiring further therapy. There was no recovery of pituitary axes postoperatively: all patients (100%) had central hypogonadism, 7/8 (88%) had central hypothyroidism, 5/8 (63%) had adrenal insufficiency, and 2/8 (25%) had diabetes insipidus, one transient and one permanent. Other surgical complications included a stroke causing right hemiparesis in one patient.

All patients were initially placed on DA therapy at doses which were stable or increased from the preoperative period. One patient (case no. 8) eventually achieved prolactin normalization while on a lower cabergoline dose than what she had been taking preoperatively, which was attributed to the decreased tumor burden. Four of 8 patients (50%) experienced significant ongoing side effects of DAs, including psychosis, hypersexuality, and headache which restricted further dose increases. Except for CSF leak, those patients who had side effects on dopamine agonist therapy preoperatively continued to have those side effects postoperatively. The two patients with significant mental health side effects had preexisting psychiatric diagnoses and were on dopamine antagonist therapy concurrently, one had to stop DA therapy entirely. No patients were treated with temozolomide. Two patients received subsequent fractionated radiotherapy, and two had subsequent craniotomy for further tumor resection (one was 2 months later, the other 3 years later).

Of the 7 patients who presented with visual deficits, 4 (57%) reported vision improvement postoperatively. No patients achieved remission off of DA therapy, but 5/8 (63%), including 3/5 patients who had preoperative DA resistance) reached remission while on ongoing DA therapy at a median time of 36 months (range 14–63 months) after initial surgery. Patients were followed for a median of 5.5 years (range 3–13 years). One patient died of unrelated causes.

## 4. Discussion

Giant prolactinomas are rare and can be mistaken for other forms of invasive brain tumor. The first-line management of prolactinoma differs from all other brain tumors; therefore, this is an important diagnosis to recognize prior to surgical intervention and tissue attainment. One barrier to diagnosis is the hook effect, which occurs when the prolactin assay is oversaturated with extremely high prolactin levels that the reading is falsely low. It is important to question mildly elevated prolactin levels and perform serial prolactin sample dilution if there is clinical suspicion for prolactinoma to provide the chance for first-line DA therapy. Newer two-step assays may overcome this effect [29].

For patients with malignant prolactinoma, temozolomide is another medication to reduce prolactin levels [27]. This treatment was not used in our cohort as all GPs were benign on pathology. It remains an area of future study to see if GP, despite being generally benign, has higher markers of proliferation such as the Ki-67 score as compared to other prolactinoma cases. Too few patients in this cohort had a Ki-67 determination to compare to prolactinomas of a smaller size.

Surgical resection of GP remains an option for patients who show resistance or intolerance to medical therapy, however, the outcomes following surgery for this patient population are not well-known as this is an uncommon treatment of a rare disease. Literature review and our surgical experience with GP reveal that surgery is rarely curative because resection is generally incomplete. The most common reason for subtotal resection is GP tumor extension and invasion, which may have close proximity to critical neurovascular structures and increase risk of surgical complications. Endoscopic endonasal approaches over the past couple of decades have allowed for more successful removal of tumors with superior extension to the third ventricle as well as those tumors that inferiorly invade the clivus. In addition, the chances for more complete GP resection continue to improve as surgical approaches and equipment, such as technological visualization, angled scopes, and improved ablative and coagulative instruments, continue to evolve. Additionally, surgeon and center experience is an important factor in determining surgical success and should be considered when weighing treatment modalities to optimize patient outcomes [30, 31].

Despite improvements in surgical techniques, there are still limitations. Lateral tumor invasion into the cavernous sinus causing internal carotid artery encasement is particularly challenging because resection can threaten cranial nerves and the internal carotid artery. Extension lateral to the optic nerve is not accessible by the transsphenoidal approach and requires a combined or staged approach with craniotomy.

Another barrier to complete resection is intratumoral fibrosis which can be tough, fibrous, and adherent to neighboring structures. Resection of a fibrotic tumor is more challenging and can lead to higher complication risks. DA therapy can variably cause these intratumoral changes with long-standing use, but the duration to develop fibrosis is unknown [32]. Reports of patients with prolactinoma of any size showed significantly lower surgical remission rates attributed to fibrotic changes for patients who had a year or more of preoperative DA therapy [33, 34]. Intratumoral fibrosis was noted in 3/6 of our DA-pretreated cases, one before a year of therapy (10, 18, and 36 months). At the same time, DA use promotes tumor shrinkage to improve the chances of successful resection [22]. DA therapy should be used preoperatively but planned surgical interventions ideally should occur before long-term DA use to avoid fibrosis as a barrier to tumor resection.

Even though all GP resections were incomplete in our series, the success of these surgical interventions is more than its surgical remission rate. For patients with CSF leak, tumor apoplexy, or acute ophthalmologic or neurologic symptoms, the focus of surgery is cranial base repair or rapid tumor decompression. For patients whose primary surgical indication is DA intolerance or resistance, the literature has been less clear on the benefit of incomplete surgical resection for GP. When looking at prolactinoma of any size, observational data showed that combined medical and surgical intervention led to the greatest improvement of visual symptoms and tumor volume reduction than either treatment alone [22, 35]. Patients who were hormonally uncontrolled preoperatively have a chance at hormonal control on similar doses of DA after surgery [20, 36–38]. In addition, targeted surgical debulking may improve the success and risk profile of adjuvant radiation techniques. From our cohort of GP cases, those who had difficulty tolerating DA therapy preoperatively had similar side effects after surgery. However, 3/5 patients who had DA resistance were able to reach normal prolactin levels after surgery (in some cases with second debulking surgery), despite being on similar or lower DA doses. This suggests that surgical reduction of tumor burden may improve DA response.

Additionally, visual deficits improved in over half of patients with GP following surgery. There was no recovery of any pituitary axis, and more patients developed hypopituitarism postoperatively, with one serious surgical complication (stroke). Most patients reached PRL normalization on ongoing DA therapy at a median of 3 years after initial surgery. This outcome data, while limited, provides prognostic information for patients and providers when considering surgical intervention for GP.

All operations were performed at our large academic center with dedicated and experienced neurosurgeons, and all diagnoses were made with histopathology confirmation. However, our study is limited by the small number of surgically-treated GP patients, even at a high-volume tertiary referral center where there were over 1,080 pituitary adenoma operations in the same time frame. Given the rarity of surgery for GP, multi-institutional or registry studies would yield clearer guidance on the optimal management of this entity.

## 5. Conclusion

Studies about the surgical management of GP are lacking. In this single-center descriptive study of all patients who underwent surgical resection of giant prolactinoma over a 15-year period, we conclude that resection is generally incomplete requiring postoperative adjuvant therapy. However, there are still advantages to incomplete surgical resection, including rapid decompression of the optic chiasm to restore vision, treatment of CSF leak or tumoral apoplexy, and reduction of tumor burden, which may allow for prolactin normalization at similar DA doses. There are high rates of postoperative hypopituitarism and surgical experience should be considered when weighing risks of complications. A majority of patients eventually achieved remission while on long-term DA therapy. This prognostic information may help set realistic expectations for patients and providers when considering surgical intervention. Further study is needed to better understand this second-line treatment of GP compared to other second-line options which include radiation therapy, somatostatin analogues, and temozolomide.

## Figures and Tables

**Table 1:** Baseline characteristics of patients with giant prolactinoma prior to treatment.

Case no	Age (years)	Gender	Race	Presenting symptoms	Initial PRL (*μ*g/L)	Preoperative central hormone deficit(s)^†^	Largest tumor dimension (cm)	Initial tumor volume (cm^3^)^†^
1	38	Male	White	Headache, weakness, visual deficit, behavior changes	N/A–not evaluated^*∗*^	N/A–not evaluated^*∗*^	5	40.0
2	53	Male	White	Weakness, visual deficit, ophthalmoplegia, behavior changes	3,482	Hypogonadism, hypothyroidism, adrenal insufficiency	7.7	109.3
3	20	Female	Black	Secondary amenorrhea, headache, visual deficit	1,111	Hypogonadism, hypothyroidism, adrenal insufficiency	6	27.3
4	39	Male	Black	Headache, weakness, visual deficit, ophthalmoplegia, behavior changes, low libido	2,498	Hypogonadism	4.6	22.8
5	26	Male	White	Headache, visual deficit, ophthalmoplegia, low libido	686	Hypogonadism	5.9	31.0
6	33	Male	White	Galactorrhea, headache, visual deficit, low libido	>13,000	Hypogonadism	5	30.6
7	47	Male	White	Headache, ophthalmoplegia, behavior change, memory loss	∼100^ǂ^	N/A–not evaluated	9	N/A–unavailable
8	51	Female	Black	Visual deficit, low libido	11,477	Hypogonadism	6	46.5

^†^Growth hormone deficiency was not tested in any patient. ^*∗*^Patient declined preoperative blood evaluation. ^ǂ^Exact value unavailable, serial dilution was not performed, falsely low prolactin level was consistent with hook effect. ^**†**^Tumor volume calculated using geometric formula, volume = ½ (*L* × *W* × *H*). PRL (prolactin).

**Table 2:** Preoperative and operative treatment course for patients with giant prolactinoma.

Case no	Preoperative DA dose and duration	PRL after initial DA (*μ*g/L)	Tumor volume after initial DA (cm^3^)^†^	Indication for surgery	Surgery type	Tumor pathology features
1	N/A	N/A	N/A	Misdiagnosis, vision loss	Craniotomy, incomplete resection	Benign, diffuse PRL stain, no co-staining
2	Cab 2.5 mg/wk for 36 mos	4,156 (19% increase)	N/A–not measured	DA resistance and intolerance, vision loss, tumor apoplexy	Transsphenoidal endoscopy, incomplete resection	Benign, diffuse PRL stain, no co-staining, Ki-67 1-2% Tumor fibrosis
3	Cab 1 mg/wk for 3 mos	262 (76% decrease)	39.4 (44% increase)	DA resistance, vision loss	Transsphenoidal using microscopy, incomplete resection	Benign, diffuse PRL stain, ACTH co-staining (No co-secretion)
4	Cab 1 mg/wk for 3 weeks	172 (93% decrease)	20.0 (12% decrease)	CSF leak, DA intolerance, vision loss	Transsphenoidal using microscopy, incomplete resection	Benign, diffuse PRL stain, GH and TSH co-staining (no co-secretion)
5	Cab 2 mg/wk for 2 weeks	364 (47% decrease)	37.2 (20% increase)	DA resistance, vision loss	Transsphenoidal endoscopy, incomplete resection	Benign, diffuse PRL stain, no co-staining
6	Cab 3.5 mg/wk for 18 mos	5,754 (>56% decrease)	27.4 (11% decrease)	DA resistance and intolerance	Transsphenoidal endoscopy, incomplete resection	Benign, diffuse PRL stain, no co-staining Tumor fibrosis
7	N/A	N/A	N/A	Misdiagnosis, vision loss	Craniotomy, incomplete resection	Benign, diffuse PRL stain, no co-staining
8	Cab 3.5 mg/wk for 10 mos	1,917 (83% decrease)	23.9 (49% decrease)	CSF leak, DA resistance and intolerance	Transsphenoidal endoscopy, incomplete resection	Benign, diffuse PRL stain, no co-staining, Ki-67 20% Tumor fibrosis

^**†**^Tumor volume calculated using geometric formula, volume = ½ (*L* × *W* × *H*). Cab (cabergoline), DA (dopamine agonist), PRL (prolactin), CSF (cerebrospinal fluid), ACTH (adrenocorticotrophic hormone), GH (growth hormone), TSH (thyroid stimulating hormone).

**Table 3:** Postoperative treatment course for patients with giant prolactinoma.

Case no	Postoperative PRL at 1 week (*μ*g/L)	Postoperative central hormone deficit(s)^†^	Postoperative PRL nadir (*μ*g/L) with DA	Time from surgery to remission (months)	DA adverse effects	Other outcomes and therapies	Duration of follow up (years)
1	4,962	Hypogonadism, hypothyroidism, adrenal insufficiency, diabetes insipidus (transient)	6 (Bromo 2.5 mg/day)	63	Worsening mental health, psychosis	N/A	5
2	368	Hypogonadism, hypothyroidism, adrenal insufficiency	2,106 (Cab 1 mg/wk)	N/A – not achieved	Psychosis, hypersexuality	Vision improved Stopped DA for adverse effects, declined radiation	5
3	63	Hypogonadism, hypothyroidism, adrenal insufficiency, diabetes insipidus	7 (Cab 1 mg/wk)	36 (0.5 from second surgery)	N/A	3 years later had another surgery: craniotomy to further debulk (growing tumor). Deceased	3
4	93	Hypogonadism, hypothyroidism, adrenal insufficiency	86 (Cab 0.5 mg/wk)	N/A–not achieved	Headache, CSF leak	Vision improved. Surgery complicated by right hemiparesis. Had radiation	6
5	184	Hypogonadism, hypothyroidism	13 (Cab 5.25 mg/wk)	15 (13 from second surgery)	N/A	2 months later had another surgery: craniotomy to further debulk	6
6	3,065	Hypogonadism, hypothyroidism, adrenal insufficiency	128 (Cab 3.5 mg/wk)	N/A–not achieved	Lightheadedness, worsening memory	Vision improved. Had radiation	5
7	11,485	Hypogonadism, hypothyroidism	5 (Cab 2 mg/wk)	14	N/A	N/A	6
8	298	Hypogonadism	1 (Cab 3 mg/wk)	43	CSF leak	Vision improved	13

^†^Growth hormone deficiency was not tested in any patient. PRL (prolactin), DA (dopamine agonist), Bromo (bromocriptine), Cab (cabergoline), CSF (cerebrospinal fluid).

## Data Availability

The data used to support the findings of this study are included within the article.
